# Adversarially Learning Occlusions by Backpropagation for Face Recognition

**DOI:** 10.3390/s23208559

**Published:** 2023-10-18

**Authors:** Caijie Zhao, Ying Qin, Bob Zhang

**Affiliations:** 1 PAMI Research Group, Department of Computer and Information Science, University of Macau, Taipa 999078, Macau SAR, China; 2 Centre for Artificial Intelligence and Robotics, Institute of Collaborative Innovation, University of Macau, Taipa 999078, Macau SAR, China

**Keywords:** occluded face recognition, deep neural network, end-to-end, adversarial learning

## Abstract

With the accomplishment of deep neural networks, face recognition methods have achieved great success in research and are now being applied at a human level. However, existing face recognition models fail to achieve state-of-the-art performance in recognizing occluded face images, which are common scenarios captured in the real world. One of the potential reasons for this is the lack of large-scale training datasets, requiring labour-intensive and costly labelling of the occlusions. To resolve these issues, we propose an Adversarially Learning Occlusions by Backpropagation (ALOB) model, a simple yet powerful double-network framework used to mitigate manual labelling by contrastively learning the corrupted features against personal identity labels, thereby maximizing the loss. To investigate the performance of the proposed method, we compared our model to the existing state-of-the-art methods, which function under the supervision of occlusion learning, in various experiments. Extensive experimentation on LFW, AR, MFR2, and other synthetic masked or occluded datasets confirmed the effectiveness of the proposed model in occluded face recognition by sustaining better results in terms of masked face recognition and general face recognition. For the AR datasets, the ALOB model outperformed other advanced methods by obtaining a 100% recognition rate for images with sunglasses (protocols 1 and 2). We also achieved the highest accuracies of 94.87%, 92.05%, 78.93%, and 71.57% TAR@FAR = 1 × 10^−3^ in LFW-OCC-2.0 and LFW-OCC-3.0, respectively. Furthermore, the proposed method generalizes well in terms of FR and MFR, yielding superior results in three datasets, LFW, LFW-Masked, and MFR2, and producing accuracies of 98.77%, 97.62%, and 93.76%, respectively.

## 1. Introduction

Face recognition (FR) is an early success story in computer vision and is achieved by deep convolution networks extracting discriminative features [1,2], sophisticated training loss formulation optimizing models [3,4,5], and large-scale training datasets [6,7]. FR methods that train with clear faces fail to maintain state-of-the-art results against real-world scenarios since there is no promise of capturing images that are free of obstructions, such as large-pose variations, poor illumination, and occlusions, in practice. A straightforward solution is training a model in occluded datasets. Unfortunately, the current existing occluded datasets do not have enough data compared with clear face datasets, causing overfitting problems [8]. Researchers have conducted extensive studies on occluded face recognition (OFR) and masked face recognition (MFR), which require the effort of hand-craft labelling the corrupted features in realistic training datasets. To alleviate the manual work, engineers are developing novel CNN-based FR methods incorporated with a gradient reversal layer (GRL) [9] to adversarially learn the occlusions using only personal identity labels while sustaining competitive results in terms of FR, OFR, and MFR compared to methods that are supervised by labels of occlusion location and individual identity.

The existing solutions to address manual annotation and the improve performance of OFR and MFR can be divided into two groups: (1) the construction of synthetic datasets by applying random occlusion generators [10,11,12,13] or masking tools [14,15]; and (2) training networks without the guidance of occlusion location by cropping the lower parts of the images for MFR [16,17] and unsupervised learning for occlusions in OFR [18]. FROM [10] and PDSN [11] utilized the same collection of realistic occlusions common in real life to add them to the original face datasets. FROM proposed an algorithm, named occluded face dataset construction, to randomly select the realistic objects as the occlusions and then occlude the faces to different occluded degrees, such as 43.09% and 52.00% of the occluded areas in the whole face. Three kinds of occlusions were applied in MSML [13] to construct the occluded training datasets, including black geometric shapes, realistic objects collected from the Internet, and synthetic face masks. INFR [12] augmented the training datasets with black rectangles as the occlusions. In terms of MFR, a masking tool, called MaskTheFace, was presented by [14] to build the masked datasets with various types of face masks, such as cloth, surgical-green, N95, etc. LPD [15] also applied this tool for converting the face datasets into the masked version. FIT [8] constructed training datasets by collecting relevant images but removed useless ones by adjusting misplaced facial data and massively scaling the original datasets. The solutions in the first group still had difficulties cooperating with real-world training datasets and would lose training images that failed to detect faces to add masks for training MFR. For MFR, the second group of methods is to remove the lower part of the images based on different cropping proportions and focus on non-masked parts such as the forehead and eyes since the mask’s location is fixed in the lower patch of the images. UPA [16] embedded an upper patch attention into the trunk CNN for extracting more meaningful features from the upper patches of the masked faces and [17] introduced a model to determine the optimal cropping in the damaged areas of the masked faces. These methods solve the manual labelling problem and improve the performance of MFR, while losing the global information of the whole face that would be useful for MFR and causing a substantial decline in the performance of FR tasks. For OFR, there are limited number of unsupervised methods. A representative method, MaskNet [18], was incorporated into the CNN architecture and assigned higher weights to the hidden units activated by the non-occluded features. It learns mask features based on the supervision of the identity labels only, but it fails to compare with the current advanced OFR methods, which have learning guidance in terms of the locations of occlusions, and does not include experiments for FR and MFR to show its generality.

The ideal model is required to extract the information from non-occluded and occluded regions, containing necessary local and global features, with effortless labelling work. The extraction of facial features is highly complicated due to various occlusions and unexpected placements occurring in facial images [19]. Inspired by the success of the GRL [9,20] in learning a large amount of labelled and unlabeled data in text and image classification, the proposed method here adversarially detects the corrupted features through the GRL optimizing the models by maximizing the loss computed for the class labels only. Occlusion is one of the most important factors in increasing the loss calculated for identity labels. This approach can train with realistic and synthetic datasets and achieve competitive results for FR, OFR, and MFR without hand-crafted labelling in occlusions.

In this paper, we propose a simple yet effective ALOB model to detect occlusions from one neural network before cleaning it from the discriminative features for the final recognition. The proposed ALOB model adopts two networks, called Identifier and Trimmer, to reveal the corrupted features caused by the occlusion in Trimmer and clean them from another feature vector generated using Identifier by feature projection purification [21]. Identifier extracts discriminative features but still contains corrupted features without careful cleaning. The feature projection purification removes the common parts between features created from Identifier and Trimmer to generate a more pure feature for the final recognition. Unlike most of the works in OFR and MFR, we do not utilize occlusion labels but train with two deep neural networks to achieve competitive results in FR, OFR, and MFR (proven by our extensive experiments). The main contribution of our work is summarized as follows:

1. We propose the ALOB model, a double-network OFR approach, which learns the corrupted and significant features via Trimmer and Identifier without annotating the occlusion location.
2. We present a new way to learn occlusion to form the feature vector from Trimmer added with GRL that optimizes the models by maximizing the loss computed for class labels only. The formed vector contains contaminated information, which should be cleaned from another feature generated from Identifier to gain the final feature vector for recognition.
3. The proposed method has been evaluated in substantial experiments on LFW [7], MFR2 [14], AR [21], and other synthetic occluded and masked datasets, achieving competitive results compared to the state-of-the-art methods supervised by occlusion labels.

The rest of the paper is structured as follows. In Section 2, we review the work related to OFR. Then, we introduce the core components of our method, Identifier and Trimmer, and the loss function in Section 3. Section 4 includes the extensive experiments compared to the advanced approaches and ablation studies. Finally, we conclude in Section 5.

## 2. Related Work

In this section, we first review some traditional works in OFR. Afterwards, the recent developments of deep learning methods in OFR are also explored and compared to our work.

### 2.1. Traditional Machine Learning-Based Methods

The traditional machine-learning-based algorithm is the earliest approach to tackling the occlusions that appeared in face recognition. Wright et al. [22] proposed the sparse-representation-based classification (SRC) in which the testing image is represented as a sparse linear combination of the training samples and then classified based on the coefficients in each class. This model is time-consuming when the training datasets are large and fails to consider errors in the sparse representation caused by more complex occlusions in practice, which is improved later by robust spare coding (RSC) [23] and structured sparse error coding (SSEC) [24]. RSC and SSEC are more powerful ways to cope with occlusions and corruption by relaxing coefficients in the sparse representation. In pursuit of having superior results by comparing each area of the face individually [22], McLaughlin et al. [25] compared face similarity by finding the largest matching area (LMA) at each point on the testing face images. Weng et al. [26] introduced robust point-set matching to the textural and geometrical information of local features extracted from the person of interest. Furthermore, Stringface [27] matched two faces through a string-to-string matching schema to find discriminative parts represented by substrings. The mentioned approaches deteriorate severely in discriminative feature representation. A novel membrane-inspired binary bat algorithm (MIBBA) [28] was introduced to enhance the representative power of local binary pattern (LBP) features from facial images fused with Gabor wavelet features. More recently, in 2020, Zhang et al. [29] obtained a more robust sparse representation than [22,23,24] by utilizing the Laplacian uniform mixture as the distribution of coding residuals. Although their works achieved sound performance in terms of benchmarks, they are limited by large-scale and complex images in practice.

### 2.2. Deep-Learning-Based Methods

To mitigate the limitations of traditional methods, Zhao et al. [30] introduced the first deep learning method to recover the occluded parts of an image through a robust LSTM–Autoencoder (RLA) model. The RLA model contains two LSTM components to convert the facial patches to latent representation via an encoder and reconstruct the faces by receiving information from the encoder. Vo et al. proposed a divide-and-conquer algorithm to address the sub-problems of degraded face reconstruction and classification by effectively applying multiple deep convolutional neural networks [31]. Another advanced texture-aware network named RFA-Net [32] employed a non-pooling residual CNN with three novel modules for finer image inpainting under the supervision of hybrid loss optimization, focusing on the semantic and texture details of the inpainting. Later, a generative adversarial network (GAN) was utilized to reconstruct faces by cooperating with a pre-trained convolutional neural network (CNN) while sustaining identity-variance features [33]. However, the image-construction method is computationally heavy and fails to contain representative features for recognition. The corruption-removal approach mitigates the issues that appeared in previous works. Removing corruption is a straightforward method, but it is hard to locate occlusions even when trained with the provided labels of occlusions in the input face images. Moreover, different models were applied such as graph convolutional networks (GCNs) and a combination of deep learning and machine learning. Vu et al. combined features extracted by neural networks and a local binary pattern (LBP) to jointly identify masked faces and published COMASK20, a masked face dataset [34]. Another well-known masked dataset, MFR2, was proposed by Anwae et al., who utilized an existing face landmark detector to extract six key-part features from the face that are necessary for its identification [14]. Albalas et al. constructed two graphs to represent the geographical similarity and correlation among facial parts and then built GCNs on fused graphs to detect the occluded faces for identifying them [35].

Most work utilized a CNN to tackle the issues. Wan et al. [18] proposed a MaskNet model, assigning lower weights to occlusions represented by nonactivated units. It is integrated into any CNN model supervised by personal labels. Using this method, it is hard to extract the most related information from the middle layers of the CNN without learning guidance, and it cannot achieve competitive results compared to other methods supervised with occlusion labels. To solve the problem, Song et al. [11] established a mask dictionary to distinguish corrupted features from occlusion-free features extracted from the top convoluted layers and then cleaned it up through multiplication with the original features. This method requires heavy computational power and an external occlusion detector to reveal the blocked regions and is improved by the most recent work from Qiu et al. [10]. Qiu et al. proposed a simple way, FROM, to dynamically decode the corrupted features learnt from the mask decoder and then assign lower weights to them through the multiplication of discriminative features. FROM is one of the best occlusion-robust face recognition methods proposed recently, and it takes the refined ResNet50 trained on the training datasets as its pre-trained model. Furthermore, the multi-scale segmentation-based mask learning (MSML) introduced by Yuan et al. [13] hierarchically extracts information from occlusions and then purifies it from multi-scale layers.

Here, most of the state-of-the-art works require the location of occlusions. Instead, the ALOB model predicts the corrupted features caused by random occlusions in the input face images through adversarial learning, maximizing the loss computed for identity labels only and then removing it from the feature vector extracted in Identifier before the final recognition. Compared to MaskNet [18] without supervision of occlusion location, our method extracts more precise information about the corrupted features since it learns occlusions from a whole network instead of gaining them from the middle of the CNN. Importantly, the ALOB model still maintains comparable performance to advanced methods with annotations of occlusions.

## 3. Method

**Figure 1:**
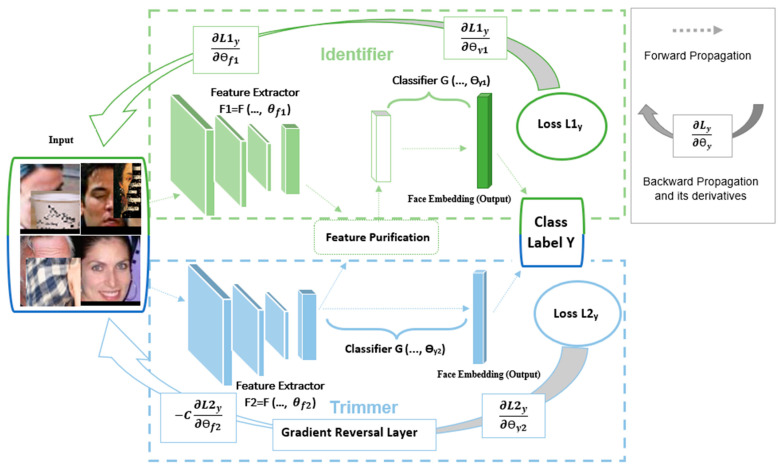
The proposed architecture contains two feature extractors for Identifier (green) and Trimmer (blue). Adversarial learning occlusion is achieved by connecting the Trimmer with the GRL [9], which multiplies the gradient with a negative constant, −C, during the back-propagation. the GRL ensures Trimmer detects features (F2) that maximize the prediction loss, ${L2}_{y}$. Identifier then extracts discriminative features (F1) and then cleans F2 from F1, which is used for final face recognition via feature projection purification (FPP) [20].

The proposed ALOB model, presented in Figure 1, is a novel double-network model trained in an end-to-end manner. It takes a min-batch consisting of clean and corrupted face images as the input and generates two opposite features for later purification. The core function of the ALOB model is to remove the features that maximize the loss calculated for identity labels to form the most purified feature vectors for the final recognition. It takes two networks, Trimmer and Identifier, to generate masked and discriminative yet corrupted features, respectively, before cleaning the masked vector from the discriminative one. Specifically, Trimmer learns the occluded features caused by the occlusions in the input face images by applying negation in the backward propagation [9]. Identifier operates like a usual neural network model to extract the prominent features; however, it removes the common parts that appeared in corrupted feature vectors through orthogonal projection [20]. Trimmer and Identifier can apply different networks as the backbones to accomplish this OFR task. Since the proposed method utilizes two networks and given the fact that the residual network (ResNet) has achieved success in most face recognition tasks, we used a relatively light model, ResNet18 [2], as the backbone for the two components in the ALOB model to illustrate the effectiveness of our models.

In the following, we first discuss the ResNet18 extractors used as the backbones in the two networks. Then, a detailed explanation of Trimmer, which extracts the corrupted features without learning guidance on occlusion location, is explored. Afterwards, Identifier with feature purification that gains the significant features and eliminates the influence of occlusions to create more discriminative features is depicted. Finally, we describe the margin-based loss functions from recent face recognition [3,4,5] that are applied in our method and include the overall training objective of the two networks.

### 3.1. ResNet Extractor

We assume that the model works with image samples $\langle \sum_{i=1}^{L}\left(x_{i},y_{i}\right)\rangle$, where $x_{i}$ represents an input face image and $y_{i}$ is an identity label corresponding to the $i^{th}$ identity in the training datasets with length L. The ALOB model consists of two subnetworks, Trimmer and Identifier, in which two of the same ResNet18 feature extractors are utilized without sharing the same parameters. These feature extractors are symbolized as $F^{m}$ and $F^{f}$. $F^{m}$ and $F^{f}$ extract the desired features, $f^{m}$ and $f^{f}$, from residual learning after receiving $x_{i}$ from the input layer, respectively. The procedure is simplified as Equations (1) and (2).

(1) $${\;f}^{m}=F^{m}(x_{i})$$

(2) $$f^{f}=F^{f}(x_{i})$$

where $f^{m}$ and $f^{f}$ are the 512-dimensionial face embedding from Trimmer and Identifier.

### 3.2. Trimmer: Gradient Reversal Layer

The principle of Trimmer is to learn a masked vector that contains the semantic information of the input clean or occluded face images that is not discriminative for the recognition task. The face classifier should not utilize corrupted features for the classification. After feature extraction, Trimmer is incorporated with the GRL [9] to negate the gradient during the back-propagation to detect the occlusion. The GRL has been applied in text and image classification for the unsupervised learning of the non-labelled features in [9]. In our case, the goal of the GRL is to adversarially learn occlusions or non-discriminative features against personal labels. Through this training module, Trimmer obtains the corrupted features from the input images.

The objective of the GRL is to reverse the gradient direction during back-propagation to optimize the model by maximizing the loss computed against the ground truth labels, i.e.,$-C\frac{\partial {L2}_{y}}{\partial \theta_{f2}}$ substitutes $\frac{\partial L2_{y}}{\partial \theta_{y2}}$ in the backward computation, as shown in Figure 1. The GRL works as an identity transform during the forward pass. Mathematically, the GRL can be formulated into Equations (3) and (4) to illustrate the schema for forward- and back-propagation [9].

(3) $$GRL\left(x\right)=x$$

(4) $$\frac{dGRL(X)}{dx}=-CI$$

where I is defined as an identity matrix and C is a hyper parameter. The 512-dimensional embedding vector, ${\;f}^{m},$ is processed via the GRL as $GRL\left({\;f}^{m}\right)={\;f}^{m\prime }$, before being fed to a face classifier, $C_{m\;},$ in Equation (5). CosFace [4], explained in Section 3.4, is used to generate the predicted labels.

(5) $$Y_{m}=C_{m}(f^{m^{\prime }})$$

(6) $${Loss}_{m}=CrossEntropyLoss(Y_{true},Y_{m})$$

${Loss}_{m}$ stated in Equation (6) is optimized by a Stochastic Gradient Descent (SGD) optimizer to enable $F^{m}$ to extract occlusions among different classes.

### 3.3. Identifier: Feature Projection Purification

The goal of Identifier is to first gain the full feature information from the input images and then clean the extracted features by projecting them into a purified semantic space for the final face recognition. Feature projection purification (FPP) [20] helps us accomplish this goal. We perform the projection from the face feature $f^{f}$ gained from $F^{f}$ into the orthogonal direction of the mask feature $f^{m}$ extracted from $F^{m}$. The orthogonal projection preserves the significant features in $f^{f}$ for the recognition task but removes the contaminated features occurring in $f^{m}$ from $f^{f},$ which cause performance degradation in the final classification. The final feature, $f_{orth\;proj}^{f},$ generated from the orthogonal projection should be made to be purer than $f^{f}$ by excluding information relevant to the mask feature $f^{m}$.

**Figure 2:**
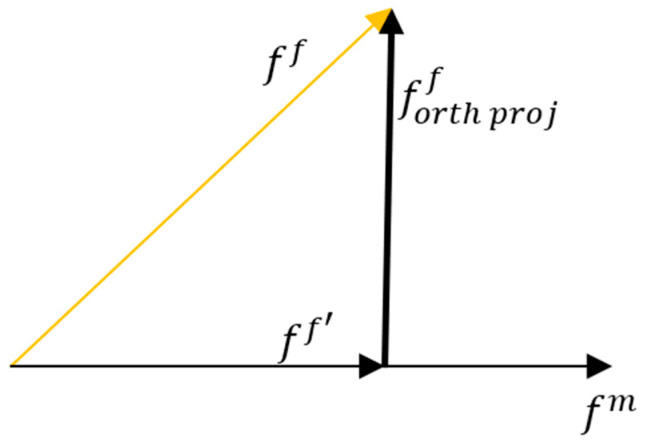
FPP projects the traditional feature vector, $f^{f},$ from $F^{f}$ onto the masked vectors $f^{m}$ from $F^{m}$ to generate the desired feature vector (in bold), $f_{orth\;proj}^{f},$ in the orthogonal direction of $f^{m}$. The desired feature vector contains purer information for the final recognition.

Figure 2 details FPP in a two-dimensional space. We first project the original feature vector, $f^{f},$ onto the masked feature vector $f^{m}$ to form $f^{f^{\prime }}$, as stated in Equation (7).

(7) $$f^{f^{\prime }}=Proj\left(f^{f},f^{m}\right)=\frac{f^{f}\cdot f^{m}}{\left|f^{m}\right|}\frac{f^{m}}{\left|f^{m}\right|}$$

where *Proj* is a projection function. $f^{f}andf^{m}$ are the 512-dimensional vectors from $F^{f}$ and $F^{m}$, respectively. The projected feature $f^{f^{\prime }}$ contains the shared features in $f^{f}$ and $f^{m}$. We obtain the orthogonal component, $f_{orth\;proj}^{f},$ of $f^{f}$ through simple vector subtraction, formulated as in Equation (8) and shown in Figure 2.

(8) $$f_{orth\;proj}^{f}=f^{f}-f^{f^{\prime }}$$

The projection from $f^{f}$ onto $f^{m}$ illustrated in Equation (7) restricts the pure vector $f_{orth\;proj}^{f}$ from containing information on $f^{f}$ instead of the trivial vectors from any other orthogonal planes to the masked feature, $f^{m}$. The pure vector $f_{orth\;proj}^{f}$ is fed into the classifier, $C_{m\;},$ as stated in Equation (9). As stated in Equation (10), the same loss function as Trimmer is applied in Identifier but is optimized by a different optimizer, the Adam optimizer.

(9) $$Y_{f}=C_{m}(f_{orth\;proj}^{f})$$

(10) $${Loss}_{f}=CrossEntropyLoss(Y_{true},Y_{f})$$

### 3.4. Loss Function

To boost the performance of face recognition, various large-margin softmax loss functions are introduced, such as CosFace [4], SphereFace [5], and ArcFace [3]. They can be effectively employed in large-scale datasets to empower more discriminative models, while Tripe Loss [36] and Contrastive Loss [37] achieve good results in small datasets. The core purpose of those loss functions is to increase the inter-class distances while decreasing the intra-class distances among all classes. We selected a large margin cosine loss, referred to as CosFace, in our model. CosFace redefines the traditional SoftMax loss in Equation (11) via L2 normalization.

(11) $$L_{softmax}=\frac{1}{N}\sum_{i=1}^{N}-\log p_{i}=\frac{1}{N}\sum_{i=1}^{N}-\log\frac{e^{f_{y_{i}}}}{\sum_{j=1}^{C}e^{f_{y_{j}}}}$$

where *N* and *C* are the number of training samples and classes, respectively. $p_{i}$ defines the posterior probability of $x_{i}$ being correctly predicted, and $f_{j}$ represents the activation of the fully connected layers with a weight vector. 

CosFace is defined as:

(12) $$L_{CosFace}=\frac{1}{N}\sum_{i=1}^{N}-\log\frac{M}{M+\sum_{j\neq i}e^{s\cos\left(\theta_{j},i\right)}}$$

$$\begin{array}{cc} w.r.t & {M=e}^{s\left(\cos\left(\theta_{y_{i}},i\right)-m\right)}\\ & \;\cos\left(\theta_{j},i\right)=W_{j}^{T}x_{i} \end{array}$$

where *m* is the margin added to control the magnitude of the cosine margin and *s* is a scaler vector. *N* is the total number of training samples. $x_{i}$ is the feature vector corresponding to the label $y_{i},$ and $\theta_{j}$ indicates the angle between the weight vector, $W_{j},$ and the feature vector, $x_{i}$. Notice that the same loss function in Equation (12) is applied in Trimmer and Identifier, where both are trained simultaneously.

### 3.5. Overall Training Objective

The overall training loss is a combination of the Trimmer loss, $L_{m},$ and the Identifier loss, $L_{f}$, as presented in Equation (13). We state the total loss as follows:

(13) $${Loss}_{total}=L_{f}+{\lambda *L}_{m}$$

where $L_{f}$ and $L_{m}$ are defined by Equation (12). λ is the weight coefficient used to control the Trimmer loss and is set to 1 in our experiments to achieve the optimization. A discussion of λ is provided in Section 4.3. Note that $L_{f}$ and $L_{m}$ are trained simultaneously but optimized by different optimizers, as suggested by [9]. The Moment SGD, with a momentum of 0.9 and a weight decay of 0.0005, optimizes Trimmer, and the Adam optimizer optimizes Identifier. Since the two losses have opposite goals, i.e., to optimize the feature extractors $F^{f}$ and $F^{m}$, the parameters between them are separated to achieve the goals and targeted in the specific subnetwork, such as making ${\;f}^{m},$ which is formed from ${\;F}^{m},$ closer to the real occlusions. The extracted features, ${\;f}^{m}$ and $f_{orth\;proj}^{f}$, are converted to $Y_{m}$ and $Y_{f}$ by the classifier, $C_{m}$, where the cosine similarity is applied to generate a one-hot prediction, and *m* and *s* are set to 0.4 and 30 for computing Equation (12).

## 4. Experiments

In this section, extensive experiments are explored to evaluate our method in realistic and synthetic datasets with various occlusions or masks only. Moreover, we specify training configurations in detail and provide a comprehensive analysis of the ALOB model using quantitative and qualitative methods.

### 4.1. Datasets and Evaluation Protocols

Our model was evaluated on various datasets, and Table 1 summarizes the statistics of the testing datasets. Detailed information about the datasets for training and testing is explored as follows.

CASIA-WebFace [38]: The CASIA-WebFace is a large-scale face dataset containing about 10,000 subjects with nearly 500,000 images crawled from the Internet. We trained our model on the WebFace dataset using its mixed version containing clean and occluded faces, following [10,11].

**Table 1:** Statistics of the training datasets and testing datasets used for performance evaluation. Types characterize whether the occluded version of the datasets is collected from real world or generated by masking tools. (* represents randomly selected pairs following [39] and “-” refers to no data required).

Names	IDs	Images	Test Pairs	Matching	Types
Training Dataset					
CASIA-WebFace	10 K	0.5 M	-	1:1	Synthetic
Testing Datasets					
LFW	5749	13,233	6000 pairs	1:1	Synthetic
AR	126	4000	-	1: N	Realistic
MFR2	53	269	800 pairs *	1:1	Realistic

Labelled Faces in the Wild (LFW) [7]: LFW includes 13,233 testing images for 5749 identities collected in an unconstrained environment. It provides 6000 testing pairs for one-to-one face verification. We evaluated our model using the standard protocols provided by [3] and presented the testing performance on 6000 test cases. 

**Figure 3:**
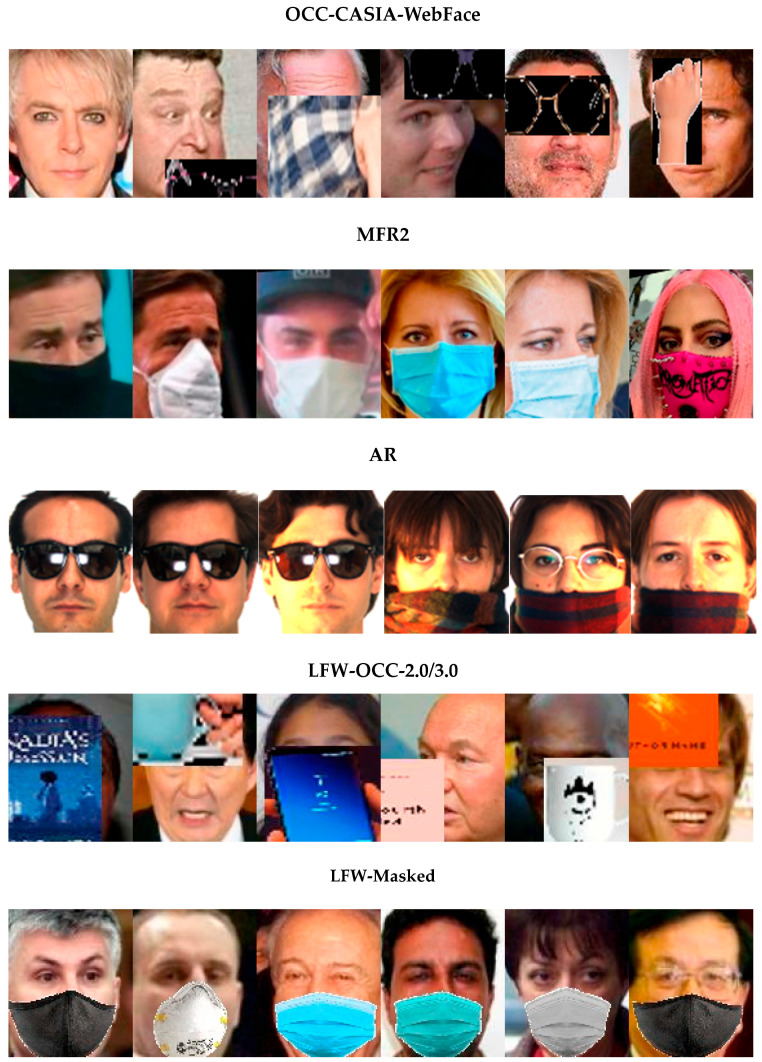
Examples of the training datasets and testing datasets in our model. The first row are images from the training dataset, OCC-CASIA-WebFace, randomly occluded by different occlusions, which are scaled from 1:0.5:5 [10]. The second and the third rows present the realistic datasets with masks in MFR2 and sunglasses and scarves in AR. The last two rows are selected from synthetic LFW datasets with 43.09% or 52% [10] occluded parts in the whole images and various types of masks.

AR Face Datasets (AR) [21]. AR is a realistic occlusion dataset used for face identification based on two protocols in our experiments. It contains 4000 face images of 126 people with differences in occlusions, illuminations, and facial expressions. Figure 3 presents some examples from AR.

Masked faces in the real world for face recognition (MFR2) [14]: MFR2 is a real-world masked face dataset with 53 subjects, including celebrities and politicians, and 269 images obtained from the Internet in total. We report the verification performance on the 800 face–mask pairs obtained from [16] by utilizing the standard protocol of face verification in [3].

**Figure 4:**
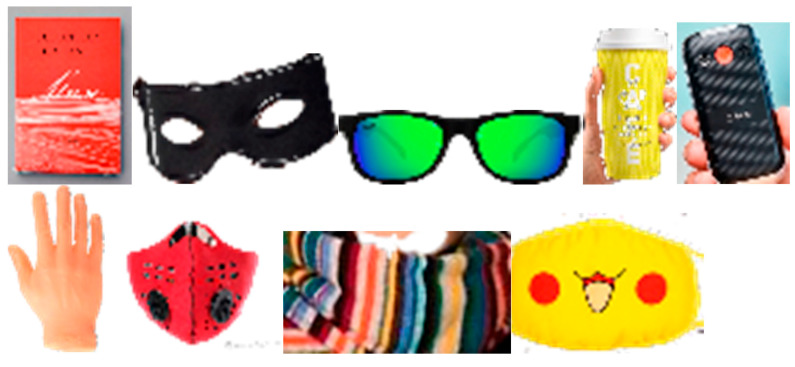
Visualizations of several occlusions utilized in the synthetic datasets provided by [10,11]. All of them are common occlusions in life encountered during face recognition, including cups, books, eyeglasses, eye masks, face masks, hands, phones, sunglasses, and scarves.

Synthetic Occluded and Masked Datasets: A face-masking tool [14] that converts face images to masked faces by randomly selecting various types of masks, such as cloth, KN95, and surgical, was utilized to construct a masked dataset, named LFW-Masked. For a fair comparison to the state-of-the-art method [16], we employed the same masking tool and selected the same types of masks stated in their paper for face verification using the standard 6000 testing pairs of LFW. We also constructed occluded datasets with various real-life occlusions, such as a cups, books, eyeglasses, eye masks, face masks, hands, phones, sunglasses, and scarves, by applying the occlusion generation algorithm from [10]. Some occlusions are visualized in Figure 4. LFW-OCC-2.0 and LFW-OCC-3.0 are generated corresponding to the occluded regions of 43.09% and 52.00% of the whole face images with randomly selected occlusions from the sample set obtained from [10]. Some synthetic samples are illustrated in Figure 4.

Evaluation Protocols: We evaluated the performance of our method on three realistic datasets and three synthetic datasets by employing two extensively used metrics. The first protocol we adopted is the accuracy metric:

(14) $$Accuracy=\frac{TP+TN}{TP+TN+FP+FN}$$

However, the accuracy metric fails to consider the scenarios when a FP is worse than a FN. The second metric is True Accepted Rate (TAR) under False Accepted Rate (FAR) and can be stated as:

(15) $$TAR=\frac{TP}{TP+FN}$$

(16) $$FAR=\frac{FP}{FP+TN}$$

where TP, TN, FP, and FN represent true positive, true negative false positive, and false negative, respectively. The performance was evaluated by a 10-fold cross-validation for one-to-one face verification, strictly following the standard protocol from [10].

Three scenarios were considered in our experiments to examine the robustness and effectiveness of the proposed model, including: (1) Face–Face (unmasked-to-unmasked), an FR task, (2) Face–Occlusion (unmasked-to-occluded), where the clean face image was utilized to identify another occluded face, and (3) Face–Mask (unmasked-to-masked), which verifies the identities of unmasked and masked faces to see whether they come from the same person.

### 4.2. Implementation Details

Preprocessing Datasets: We first aligned and cropped face images by utilizing an MTCNN [40] to detect facial landmarks (two eyes, a nose, and two corners of the mouth), which resized the face images into 112 × 112 pixels. This was carried out since the raw images contain lots of background information, impairing the performance of the models [41]. Finally, the 112 × 112 images were resized to 112 × 96 pixels through a similar transformation in terms of alignment and cropping before being normalized to [−1.0, 1.0] in training and testing following [4,10,11]. We employed the random occlusion generator [10], which masked the final 112 × 96 face images.

Training Configuration: For a fair comparison to other advanced methods, we applied ResNet18 or LightCNN-9 (L9) [42] as the backbones of the ALOB model. Note that Identifier and Trimmer cooperated with identical feature extractors in all our experiments, with different optimizers stated in Section 3.5. All the models were trained for 21 epochs with a batch size of 128 without any pre-trained models via one NVIDIA T4 GPU in the Linux system. The framework for our model was written in the PyTorch using Python. We set the initial learning rate to 0.01 and 0.001 for training ResNet18 and L9 as the backbones in the various experiments, respectively. The learning rates were divided by 10 at epoch 10. The training dataset was a mixed dataset with clean and occluded face images from CASIA-WebFace and a selection ratio of 1:2 for the whole training process, following the setting used in [10]. The scale was randomly chosen as 1:0.5:5, that is (1, 1.5, 2, 2.5, etc.) to determine the size of the occluded parts in the images. We named the training dataset OCC-CASIA-WebFace. Data augmentation, such as random horizontal flips and changing the brightness, contrast, saturation, and hue, was also applied during the training process. 

Baseline Settings: In our experiments, we selected two networks as the baselines, ResNet18 and L9. As illustrated in Figure 1, although we trained the two subnetworks, only cleaned features extracted from Identifier were utilized for the final recognition without adding more features generated from another backbone. Here, the baselines are a single-network framework. Compared to the ALOB model, we designed two baselines as the typical feature extraction to showcase that the proposed method removes corrupted features while keeping the useful ones.

Baseline—ResNet18: We applied ResNet18 as the feature extractor (i.e., Identifier without FPP, as shown in Figure 1). The backbone is trained on OCC-CASIA-WebFace using the CosFace loss for 21 epochs with an initial learning rate of 0.01.

Baseline—L9: Another baseline, L9, was employed as the feature extractor. The training set was the same as the baseline ResNet18, but we changed the initial learning rate to 0.001. Compared to ResNet18, L9 is a lighter model, allowing us to assign a small learning rate to train L9 as the feature extractor.

### 4.3. Test Performances

As stated before, the proposed model was trained on OCC-CASIA-WebFace datasets with the weighted loss function shown in Equation (13). Here, we defined two baselines for a comparative analysis and utilized the same type of backbones as the main state-of-the-art models for a fair comparison. Some other factors were considered in our experiments such as the training datasets and seen occlusions (trained occlusions), shown in Table 2. We compared our model to the most recent and advanced OFR methods, FROM [10] and MSML [13], on occluded versions of LFW and AR, respectively. Both methods used the OCC-CASIA-WebFace dataset for training, including the same occlusions that appeared in the testing cases. Our methods with lighter backbones and without manual labelling work can still outperform FROM and MSML requiring annotating labels. Furthermore, we also compared ALOB to other OFR methods with and without guidance on occlusion location, such as PDSN [11] and MaskNet [18], and our method surpassed all of them for the AR datasets. For LFW-OCC-2.0 and 3.0, we tested ALOB using two metrics and gained higher results than FROM trained using labels of occlusions and personal identities. We summarize the detailed information in Table 3 and Table 4. To evaluate the generality of our model in the Face–Face and Face–Mask test cases shown in Table 5, we compared the performance of our model with the recently proposed MFR methods, LPD [15] and UPA [16]. ALOB is not targeted for MFR, but it generalizes well in MFR tasks, even compared to MFR methods.

We first evaluated our model on AR for face identification corresponding to two protocols with two occlusions, sunglasses (sg) and a scarf, and included the results in Table 3. The gallery set is used for identifying the person in the problem set, which contains six images per person in protocol 1 and only one photo per person in protocol 2. The accuracy can be calculated separately in terms of different occlusions. The same types of sunglasses and scarves are included during training for MSML and ALOB. MSML incorporates LightCNN-29 (L29) [42] as its backbone, which includes residual blocks in the architecture. Table 3 illustrates that the ALOB model trained in ResNet18 consistently surpasses other methods on AR. We also trained our model in L9, a lighter framework than L29, without residual blocks. ALOB-L9 obtained higher accuracies than MSML by more than 0.2% in the problem set of sg (protocols 1 and 2) and scarf (protocol 2), respectively. The performance of the baseline L9 was worse than that of L29 reported in MSML, but our method can attain a better performance than MSML. On average, the proposed method improved by more than 1.5% and 5.5% in terms of accuracy for sg and gained around 1% and 3.5% in accuracy for the scarf problem set, corresponding to two different protocols, compared to Seg-DGDNet [43], PDSN [11], and RPSM [26]. In terms of protocol 1, ALOB remarkably outperformed other OFR methods, which only reported data on protocol 1 [22,44,45,46] by achieving almost 100% accuracy (more than a 2% improvement in ALOB with different backbones). OFR models with only results from protocol 2 [25,27,41] obtained around 94% accuracy on the scarves datasets, while ALOB attained better performance with an accuracy of 99%. Compared to MaskNet [18], trained only under the supervision of identity labels, that is, the same as our method, ALOB with different backbones achieved higher accuracies, significantly surpassing more than 9% and 3% in the sg and scarves datasets under protocol 1. Furthermore, our models outperformed the baselines in all test cases, which implies a cooperation between the GRL and FPP to remove the corruption from the extracted features instead of the useful ones for the classification.

We conducted more challenging experiments for face verification in the synthetic occluded datasets shown in Table 4. Two versions of occluded LFW, LFW-OCC-2.0, and LFW-OCC-3.0 were constructed by the random occlusion generator [10], representing 43.09% and 52% occluded parts in LFW images, respectively. The models were evaluated in terms of their accuracy and another strict metric, the TAR, which was computed with the FAR set to $1\times 10^{-3}$. Not surprisingly, the baseline ResNet18 was significantly improved by more than 1.5% in accuracy and 10% in the TAR for both datasets. Our model obtained better results than FROM in terms of accuracy and the TAR. The ALOB method and FROM both constructed a 512-dimensional face embedding for the final face classification. However, the ALOB attained more than 0.1% and around a 1.5% improvement in both datasets regarding accuracy and the TAR. The proposed method sustains competitive performances in more complex datasets.

Experiments in Masked Datasets: To evaluate the effectiveness and robustness of the ALOB model, we employed our model for FR and MFR, including realistic and synthetic types of face masks. We compared the ALOB model to other models targeted for OFR and MFR for all the clean and masked face datasets shown in Table 5. We retrained our model using the same occlusions stated in the UPA for fair a comparison. In our training, masks were randomly assigned to the faces, while a masking tool was applied to mask the faces in the UPA. The proposed method still shows its superiority under training through the random masking of faces. The proposed model achieves the best accuracy of 93.76% on MFR2 (Face–Mask) compared to all the OFR and MFR methods. The ALOB model also gained the best performance on LFW-Masked, improving by 0.02% compared to the UPA, which obtained 97.60% in accuracy. Our model outperformed other state-of-the-art methods for OFR, such as PDSN [11], INFR [12] DFM [47], and the MFR model LPD [15], by at least 4.5% on average for OFR methods and 3% for the MFR model in LFW-Masked, respectively. We also conducted experiments for our model in terms of FR using the same model targeted for MFR to evaluate its generalizability. It is seen in Table 5 that ALOB exceeded other methods and the baseline ResNet18. Table 6 summarizes the performance of the ALOB model with the stricter metric, the TAR. When compared to the baseline ResNet18 in terms of accuracy and the TAR, our model has an upgradation performance for FR, revealing that Trimmer detects harmful or confusing features that maximize the loss calculated against the identity labels and preserves the significant features in Identifier, as we stated before.

### 4.4. Discussion of λ

The weight coefficient, λ, in Equation (13) controls the trade-off between the face recognition loss in Identifier, $L_{f}$, and the loss for corrupted feature prediction in Trimmer, $L_{m}$. We designed experiments to find the optimal value of λ, as demonstrated in Table 7. It was tested with $\lambda \;\epsilon \left[0,\;1\times 10^{-3},\;1\times 10^{-2},\;1\times 10^{-1},\;0.5,\;1,\;2,\;3\right]$ under the same training configuration stated in Section 4.2 on the LFW-OCC-2.0, LFW-OCC-3.0, and MFR2 datasets. Small values suppress the performance for occlusion prediction, while large values cause the model to emphasize occlusion prediction instead of face classification. Obviously, the optimal value is 1.0.

### 4.5. Ablation Study

Various ablation studies were conducted in this subsection to explore the impact of different parts of our model. We chose occluded and real-world masked datasets, LFW-OCC-2.0, LFW-OCC-3.0, and MFR2, as the testing datasets used for the ablation experiments. The removed module is specified in the model names, where ALOB -G/-F/-G-F represents GRL/FPP/both GRL and FPP removed. The performance of the models was evaluated by the two metrics defined in Section 4.1. Note that all the models utilized ResNet18 as the backbone and the same configuration as the ALOB model stated in Section 4.2.

Analysis of the GRL and FPP: The experimental results for examining the GRL and FPP individually (ALOB-G and ALOB-F) under different test scenarios are illustrated in Table 8. In ALOB-F, we replaced $f^{f}-f^{f^{\prime }},$ defined in Section 3.3, by $f^{f}-f^{m}$, which is a simple subtraction between features that was extracted from Identifier and Trimmer. Whether or not the GRL or FPP is removed, the performance drops in terms of accuracy and the TAR when the FAR is set to $1\times 10^{-3}$. Here, there is severe degradation in the TAR for all the testing datasets, decreasing by nearly 6% on average when the GRL is removed or FPP is replaced by a simple subtraction between two features, $f^{f}$ and $f^{m}$. These results showcase the importance of each component in our models, with the absence of one of these submodules leading to a significant degradation in performance. 

Analysis of the proposed method: We also designed experiments for comparing our model with other combinations of two subnetworks to show the superiority of our model. The GRL and FPP were removed, and the summation of two features was utilized to form the 512-dimensional face embedding for the final classification. The two feature vectors contained discriminative information in the ALOB-G-F (sum), such that its sum augmented the prominent features. It is seen in Table 7 that the proposed model outperforms the ALOB-G-F (sum) in terms of the two metrics. Another model, ALOB-G-F (concat), concatenates two features to generate a 1024-dimensional face embedding, but we still compare it to our 512-dimensional face embedding in the ALOB model. Although ALOB-G-F (concat) contains more parameters for face recognition, our model can sustain a slightly higher accuracy in all the datasets. There is still a gap between the proposed model and the ALOB-G-F (concat) regarding the TAR, decreasing by roughly 3% in terms of feature concatenation. These experimental results meet our expectations. With FPP, features relevant to the masked feature $f^{m}$ are excluded from $f^{f}$. This way, the output face embedding is the most discriminative was to describe images. These two experiments demonstrate the effectiveness of the design of our model to tackle the issues of OFR.

### 4.6. Visualization of the Proposed Model

#### 4.6.1. Feature Visualization

**Figure 5:**
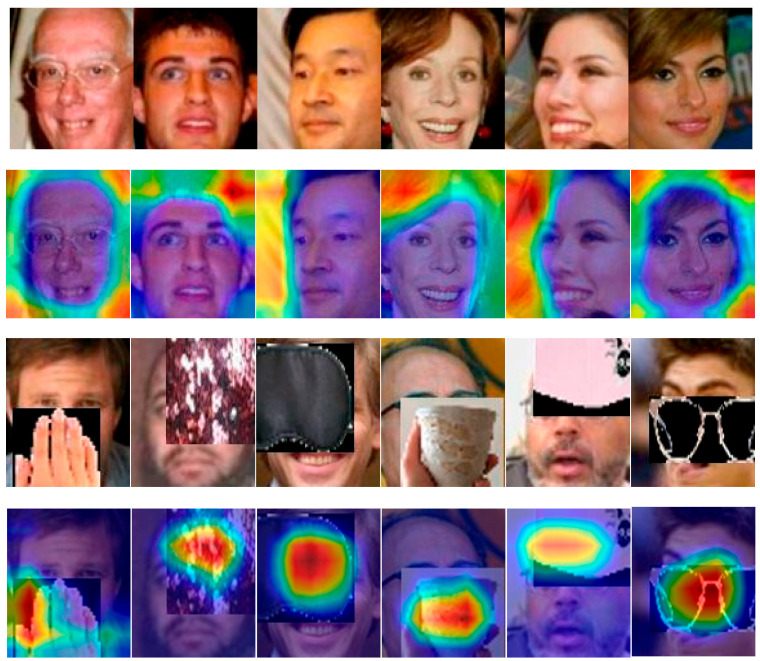
Visualizations of the predicted harmful features extracted from Trimmer in clean and occluded images. We selected the second last convolution layer in the ResNet18 as the targeted layer for computing the CAM [48]. The higher weights are assigned with brighter color.

To demonstrate the goal of Trimmer, detecting the harmful features for occluded and clean face classification, we utilized GradCAM++ and GradCAM [48] to visualize the feature maps produced from the second-to-last convolution layer. The clean face images were selected from the original LFW datasets. The occluded samples were selected from LFW-OCC-2.0 and LFW-OCC-3.0. Visualizations of the features learned by Trimmer are presented in Figure 5. CAM assigns brighter colors to obvious features with higher weights in the feature map. Figure 5 confirms that Trimmer can extract the corrupted features. Specifically, brighter colors signify that higher weights are assigned in the surrounding areas of the face, such as the hairs in the clean face images, and obvious colors are located in corrupted features in terms of the occluded images.

#### 4.6.2. Feature Space Visualization

**Figure 6:**
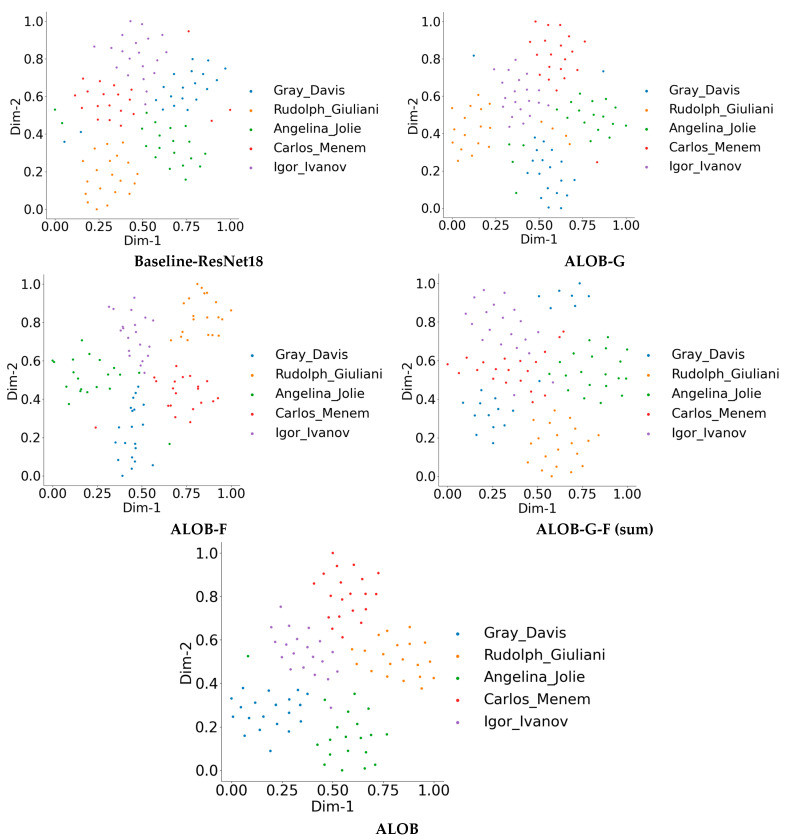
Visualizations of feature distribution by converting 512 dimensions to 2 dimensions with t-SNE [49]. The features were extracted from five randomly picked people from LFW-OCC-2.0, and each class contains 20 samples. Zoom in for better visualization.

To explore the improvement of our proposed method in coping with facial occlusions in depth, we also qualitatively compared our model to the other models defined in Section 4.4 for the ablation experiments. Five different identities were selected from LFW-OCC-2.0 with 20 sample images, and each model generated 512-dimensional face embedding for the final face recognition. T-SNE [49] converts the 512D feature space to a 2D feature space for convenient visualization, and the results are presented in Figure 6. Note that we trained all the presented models on OCC-CASIA-WebFace, including the baseline ResNet18. Compared to other models, the proposed model enhances intra-class compactness and inter-class discrepancy and reduces the ambiguities in the decision boundaries among different classes.

## 5. Conclusions

This work presents a simple yet efficient double-network framework for occlusion-robust face recognition, which consists of contrastively learning the corrupted features against the identity labels only and then removing the harmful information from the final face embedding for the final classification. The ALOB model tackles the problem of having to label the locations of occlusions by hand in a novel way and still sustains the performance of FR, MFR, and OFR compared to existing methods that function under the guidance of occlusion learning. Furthermore, we provide quantitative and qualitative experiments to illustrate the effectiveness and generality of our approach to addressing face recognition. To further improve the performance of OFR, we will consider incorporating our model into pretrained models to initially assign better feature extractors in Identifier and Trimmer. A straightforward choice is using a deep neural network such as ResNet50 trained on the targeted objects as a good initialization for each subnet.

## Figures and Tables

**Table 2:** The training datasets, including the occlusions from the state-of-the-art methods compared in our experiments.

Models	Training Datasets	Occlusions in Training
MSML(L29)	OCC-CASIA-WebFace	geometric shapes, realistic objects (sunglasses, scarf, face mask, hand, etc.), masks
FROM(ResNet50)	OCC-CASIA-WebFace	sunglasses, scarf, face mask, hand, eye mask, eyeglasses, book, phone, cup
UPA(ResNet28)	CASIA-Webface-Masked	face masks (cloth, surgical-blue, surgical-green, KN95, surgical-white)

**Table 3:** Face identification results in realistic occluded AR datasets (* refers to the data reported in [13], and names without * signifies data reported in the original papers. “-” represents the missing data in their reported papers). In addition, 1/2 refers to protocol 1 and 2, correspondingly. The best performance is highlighted as bold.

Models Accuracy	AR (sg 1/2)	AR (scarf 1/2)
Baseline-L9	97.5/94.72	97.64/89.03
Baseline-ResNet18	100/99.58	99.96/99.30
ALOB-L9 (ours)	**100/99.03**	99.86/98.61
ALOB (ours)	**100/99.72**	**100/99.58**
* L29	98.02/96.44	98.78/96.76
Seg-DGDNet (2023) [43]	99.71/98.73	100/99.03
MSLM (2022) [13]	99.84/98.80	100/99.37
PDSN (2019) [11]	99.72/98.19	100/98.33
RPSM (2016) [26]	96/84.84	97.66/90.16
DDF (2020) [40]	-/98	-/94.10
* LUMIRC (2020) [29]	97.35/-	96.7/-
MaskNet (2017) [18]	90.90/-	96.70/-
NMR (2017) [44]	96.9/-	73.5/-
LMA (2016) [25]	-/96.30	-/93.70
MLERPM (2013) [45]	98.0/-	97.0/-
SCF-PKR (2013) [46]	95.65/-	98.0/-
StringFace (2010) [27]	-/82.00	-/92.00
SRC (2008) [22]	87.0/-	59.50/-

**Table 4:** Face verification results in synthetic occluded datasets (* refers to data obtained from running the provided model [10] by ourselves, and data without * represent the reported results in FROM). The best performance is highlighted as bold.

Models $\mathrm{Accuracy}/\mathrm{TAR}@\mathrm{FAR}\;=\;1\times 10^{-3}$	LFW-OCC-2.0 (Face–Occlusion)	LFW-OCC-3.0 (Face–Occlusion)
Baseline-ResNet18	93.40/64.00	90.08/61.73
ALOB (ours)	**94.87/78.93**	**92.05/71.57**
FROM (2022) [10]	94.70/76.53	91.60 */70.27 *

**Table 5:** Face verification accuracies (%) in Face–Face and Face–Mask test cases. (Testing pairs in MFR2 are provided in UPA, and “-” represents the missing data in their reported papers). The best performance is highlighted as bold.

Models Accuracy	LFW (Face–Face)	LFW-Masked (Face–Mask)	MFR2 (Face–Mask)
Baseline-ResNet18	98.27	96.80	91.76
ALOB (ours)	**98.77**	**97.62**	**93.76**
UPA (2022) [16]	98.02	97.60	92.38
LPD (2020) [15]	94.82	94.28	88.76
INFR (2019) [12]	97.66	93.03	90.63
PDSN (2019) [11]	-	86.72	-
DFM (2018) [47]	-	92.88	-

**Table 6:** Comparison of TAR @ FAR = $1\times 10^{-3}$ between the baseline ResNet18 and ALOB in Face–Face and Face–Mask test cases. The best performance is highlighted as bold.

Models $\mathrm{TAR}@\mathrm{FAR}\;=\;1\times 10^{-3}$	LFW (Face–Face)	LFW-Masked (Face–Mask)	MFR2 (Face–Mask)
Baseline-ResNet18	88.40	83.07	56.11
ALOB	**95.90**	**89.63**	**66.83**

**Table 7:** Face verification accuracies and TAR impacted by different weight coefficients in Equation (13) on three datasets. The best performance is highlighted as bold.

λ $\mathrm{Accuracy}/\mathrm{TAR}@\mathrm{FAR}\;=\;1\times 10^{-3}$	LFW-OCC-2.0 (Face–Occlusion)	LFW-OCC-3.0 (Face–Occlusion)	MFR2 (Face–Mask)
λ _0_	92.45/66.10	91.82/67.43	91.89/57.11
λ _0.001_	94.22/72.13	91.28/70.27	91.76/52.34
λ _0.01_	94.38/77.13	91.88/69.77	92.01/57.33
λ _0.1_	94.13/74.17	90.33/58.47	92.51/57.86
λ _0.5_	92.32/66.67	89.85/59.33	90.14/58.83
λ _1.0_	**94.87/78.93**	**92.05/71.57**	**93.76/66.83**
λ _2.0_	93.78/73.70	91.65/66.17	92.01/60.33
λ _3.0_	93.67/67.4	91.37/65.77	92.13/60.85

**Table 8:** Ablation studies. The first two experiments investigated the effectiveness of the GRL or FPP by removing them in the ALOB-G and ALOB-F, respectively. Two modules, the GRL and FPP, were removed in the last two experiments, where the features from Trimmer and Identifier are summed in ALOB-G-F (sum) or concatenated in ALOB-G-F(concat). The best performance is highlighted as bold.

Models $\mathrm{Accuracy}/\mathrm{TAR}@\mathrm{FAR}\;=\;1\times 10^{-3}$	LFW-OCC-2.0 (Face–Occlusion)	LFW-OCC-3.0 (Face–Occlusion)	MFR2 (Face–Mask)
ALOB-G	92.45/66.10	91.82/67.43	91.89/57.11
ALOB-F	94.62/74.27	90.35/64.10	91.51/60.59
ALOB-G-F (sum)	94.38/70.47	92.02/70.10	91.51/62.01
ALOB-G-F (concat)	94.45/74.77	91.77/70.13	92.01/63.84
ALOB	**94.87/78.93**	**92.05/71.57**	**93.76/66.83**

## Data Availability

Publicly available datasets (CASIA-WebFace, LFW, AR, LFW-OCC-2.0, LFW-OCC-3.0) can be found here: https://github.com/haibo-qiu/FROM/tree/main#data-preparation, accessed on 10 September 2023. Publicly available dataset, MFR2, can be found here: https://github.com/aqeelanwar/MaskTheFace#mfr2---masked-faces-in-real-world-for-face-recognition, accessed on 10 September 2023.
